# Study on the relationship between the pathogenic mutations of SLC26A4 and CT phenotypes of inner ear in patient with sensorineural hearing loss

**DOI:** 10.1042/BSR20182241

**Published:** 2019-03-22

**Authors:** Lihua Wu, Yunliang Liu, Jianman Wu, Sheng Chen, Shupin Tang, Yi Jiang, Pu Dai

**Affiliations:** 1 Department of Otolaryngology, Head and Neck Surgery, Nanfang Hospital, Southern Medical Uiversity, Guangzhou, Guangdong, P.R. China; 2 Department of Otolaryngology, Head and Neck Surgery, Shengli Clinical Medical College of Fujian Medical University, Fujian Provincial Hospital, Fuzhou 350001, Fujian, P.R. China; 3 Department of Otolaryngology, Fujian Provincial Maternity and Children Hospital, Fuzhou 350001, Fujian, P.R. China; 4 Department of Radiology, Shengli Clinical Medical College of Fujian Medical University, Fujian Provincial Hospital, Fuzhou 350001, Fujian, P.R. China; 5 Department of Ultrasound, Shengli Clinical Medical College of Fujian Medical University, Fujian Provincial Hospital, Fuzhou 350001, Fujian, P.R. China; 6 Department of Otolaryngology, Head and Neck Surgery, PLA General Hospital, Beijing 100853, P.R. China

**Keywords:** CT phenotype, Sensorineural hearing loss, SLC26A4 gene mutation

## Abstract

To investigate the possible association of pathogenic mutations of SLC26A4 and computerized tomography (CT) phenotypes of inner ear, and explore the feasibility of using the method of gene sequence analysis. A total of 155 patients with bilateral hearing loss carrying SLC26A4 gene mutations were further subjected to high-resolution temporal bone CT and thyroid B ultrasound tests. The potential relationship between the pathogenic mutations of gene and the CT phenotypes were analyzed. As a result, 65 patients (41.9%, 65/155) carried SLC26A4 gene mutations, and 27 cases were detected with pathogenic mutations of SLC26A4 where IVS7-2A>G (55.6%, 15/27) was the most common pathogenic mutation. Amongst them, 19 patients carrying bi-allelic SLC26A4 mutations were all confirmed to have inner ear malformation by CT scan including four cases of enlarged vestibular aqueduct (EVA) and 15 cases of Mondini dysplasia (MD). However, there was only one in eight cases of single allele pathogenic mutation who was confirmed to have EVA by CT scan. Further, only one patient with EVA was confirmed to be slightly higher of total T3 than normal by thyroid ultrasound scan and thyroid hormone assays. These findings suggested that CT detection and *SLC26A4* gene detection are efficient methods to diagnose EVA, which can complement each other. Also, the bi-allelic pathogenic mutations of SLC26A4 are more likely to induce inner ear malformation than single allele pathogenic mutation.

## Introduction

Congenital hearing impairment is the most common neurosensory disorder in humans, which is usually associated with abnormalities of inner ear structures. The preferred diagnostic method for inner ear malformation is computerized tomography (CT) examination, because it can make a clear diagnosis even of some severe inner ear malformations [1]. However, there still exists misdiagnosis or miss while in some cases of minor lesions, such as incomplete separation, cochlea axis and screen area malformations, due to the lack of high-resolution CT equipment and experienced imaging diagnostic physicians [2]. Therefore, it is meaningfull to develop an alternative or complementary approach to improve the diagnosis of inner ear malformation.

Generally, more than half of all cases of hearing impairment are caused by mutations in genes related to the hearing process [3]. Sensorineural hearing loss (SHL) is considered a monogenic disease in which the inactivation of one of the genes is enough to cause the disease [4]. It was reported that mutation in SLC26A4 was not only associated with the common causes of non-syndromic SHL in many ethnic populations [5–9], also biallelic mutations in SLC26A4 could cause Pendred syndrome (PDS), the most common form of syndromic deafness, which accounts for approximately 10% of hereditary hearing impairment [7]. Although mutation analysis of SLC26A4 have been applied for diagonsis of PDS and non-syndromic hearing loss [3,10–13], SLC26A4 mutations cannot be detected in approximately one-third of patients with enlarged vestibular aqueduct (EVA), whereas only one mutant SLC26A4 allele was identified in another third [14–16]. It was suggested that there were still challenges in accurate clinical diagnosis of inner ear malformation in deafness patients.

Here, in order to improve the diagnosis of inner ear malformation in patients with SHL, we explored the relationship between the pathogenic mutation type of GJB2 SLC26A4 and CT phenotype based on sequencing all the exons of *SLC26A4* gene of 155 deaf patients, and further analyzed the correlation of *SLC26A4* mutations with thyroid function, and thyroid B ultrasound.

## Materials and methods

### Patients and DNA samples

A total of 155 unrelated patients with hearing impairment (152 students and 3 teachers) from Xiamen Special Education School in Fujian province were included in the present study. This cohort of patients consisted of 93 males and 62 females ranging from 5 to 36 years with an average of 13.4. These patients included 153 Han and 2 She races. Hearing tests demonstrated that the level of hearing loss was severe to profound in all cases. There were 25 patients whose families had more than one other family member with a hearing impairment. No family had a consanguineous marriage except that one patient’s parents were cousins. The protocol for this investigation was performed with the approval of the ethics committees of the Chinese PLA General Hospital and Fujian Provincial Hospital. The notification of deafness gene detection was performed a half month prior. We obtained informed consent and individual information questionnaire, including name, age, address, family history, health records of the mother during pregnancy, and a clinical history of the patient, such as infections, possible head or brain injury, and the use of aminoglycoside antibiotics from all patients and guardians who volunteered to participate in the present study prior to blood sampling. All subjects underwent hearing tests and medical examinations.

### Mutational analysis

DNA was extracted from peripheral blood leukocytes using a commercially available DNA extraction kit (Watson Biotechnologies Inc., Shanghai, China). The genomic DNA of the affected individuals was examined for SLC26A4 mutations based on the previously described studies [7,11,17]. The coding exons plus approximately 50–100 bp of the flanking intron regions of SLC26A4 were amplified by PCR, and Sanger sequencing was used to determine the sequences in all patients. According to the manufacturer’s manual, the results were analyzed using an ABI 3100 DNA sequencing machine (ABI, Foster City, CA, U.S.A.) and the ABI 3100 Analysis Software (ver. 3.7 NT). Data analysis was performed using SPSS 20.0 software. After sequencing all the 20 exons of *SLC26A4* gene of 155 patients, there were 65 cases detected with *SLC26A4* gene mutation, of which 63 were further examined.

### Assays of CT scan and thyroid function

The 63 patients with *SLC26A4* mutation were further subjected to high-resolution temporal bone CT scan for the diagnosis of EVA or inner ear malformation based on the criteria of a diameter greater than 1.5 mm between the outer vestibular aqueduct and the vestibule of the total port foot or the midpoint of the isthmus [18]. Thyroid B ultrasound and thyroid function tests were applied for evaluation of PDS in the patients with positive SLC26A4 mutations or variants. These tests were completed by two specific attending physicians separately in Fujian Provincial Hospital. The statistical significance of the difference was analyzed by Fisher’s exact test using SPSS 20.0 software.

## Results

### Genetic test

As shown in Table 1, sequence analysis of SLC26A4 in these 155 students and teachers with hearing impairment identified that 19 patients carryied two confirmed pathogenic mutations’ alleles, and 8 patients carried one mutant allele, that the pathogenic mutation rate was 17.4% (27/155). A total of 17 different mutations (IVS7-2A>G, 1079C>T, 916-917insG, 1738-1739delA, 1229C>T, 1692-1693insA, 2086C>T, 2168A>G, 1472T>C, 1595G>T, IVS16-6G>A, 1764-1765insAGGAAAATA, 2007C>G, 1336C>T, 754T>C, 147 C>G, IVS11+47T>C) were identified as pathogenic variants according to ‘*Standards and Guidelines for the Interpretation of Sequence Variants: A Joint Consensus Recommendation of the American College of Medical Genetics and Genomics and the Association for Molecular Pathology*’. Amongst them, 15 of 27 patients were comfirmed to have IVS7-2A>G mutation (55.6%, 15/27), which indicated that IVS7-2A>G was the most common aberrant splice site alteration in SLC26A4 mutation of our patient cohort in Xiamen. There was also one patient (XT1047) carrying a novel unclassified variant (147C>G), which was likely pathogenic due to its evolutionary conservation and conserved amino acid change. Specifically, the mutation rate of 2009T>C (p.V670A) was was 1.29% (2/155) which was not found in 1668 EVAS patients from our laboratory before the present study [19]. We evaluated the pathogenicity of this mutation by SIFT and Polyphen-2, and the results suggested ‘tolerant’ with a SIFT score of 0.05 and ‘possibly damaging’ with a Polyphen-2 score of 0.873 by each, thus, a conclusion still cannot be reached.

**Table 1:** Genotypes and CT phenotypes of the 64 patients with *SLC26A4* gene mutations at Xiamen City Special Education Schools of deafness

No.	Gender	Age (years)	Allele 1			Allele 2			TBCT	TBUS	TF
			Nucleotide change	Mutation type or amino acid change	Characterization of variant	Nucleotide change	Mutation type or amino acid change	Characterization of variant			
XT117	F	18	c.754T>C	p.S252P	Pathogenic	c.1738_1739delAA	FS580, P606*	Pathogenic	IEVA	Nl	Nl
XT020	M	11	c.916-917insG	FS306, P329*	Pathogenic	c.2168A>G	p.H723R	Pathogenic	IEVA	Nl	Nl
XT022	F	9	c.IVS7-2A>G	Alteration of splicing sites	Pathogenic	c.IVS7-2A>G	Alteration of splicing sites	Pathogenic	MD	Nl	Nl
XT102	M	15	c.IVS7-2A>G	Alteration of splicing sites	Pathogenic	c.IVS7-2A>G	Alteration of splicing sites	Pathogenic	MD	Nl	Nl
XT110	F	8	c.IVS7-2A>G	Alteration of splicing sites	Pathogenic	c.IVS7-2A>G	Alteration of splicing sites	Pathogenic	MD	Nl	Nl
XT162	F	8	c.IVS7-2A>G	Alteration of splicing sites	Pathogenic	c.IVS7-2A>G	Alteration of splicing sites	Pathogenic	MD	Nl	Nl
XT149	M	15	c.IVS7-2A>G	Alteration of splicing sites	Pathogenic	c.IVS7-2A>G	Alteration of splicing sites	Pathogenic	MD	Nl	Nl
XT063	F	11	c.IVS7-2A>G	Alteration of splicing sites	Pathogenic	c.1079C>T	p.A360V	Pathogenic	MD	Nl	Nl
XT066	M	15	c.IVS7-2A>G	Alteration of splicing sites	Pathogenic	c.1079C>T	p.A360V	Pathogenic	MD	Nl	Nl
XT147	F	18	c.IVS7-2A>G	Alteration of splicing sites	Pathogenic	c.1079C>T	p.A360V	Pathogenic	MD	Nl	Nl
XT010	M	11	c.IVS7-2A>G	Alteration of splicing sites	Pathogenic	c.2086C>T	p.Q696*	Pathogenic	MD	Nl	Nl
XT012	F	8	c.IVS7-2A>G	Alteration of splicing sites	Pathogenic	c.2086C>T	p.Q696*	Pathogenic	MD	Nl	Nl
XT148	M	13	c.IVS7-2A>G	Alteration of splicing sites	Pathogenic	c.1336C>T	p.Q446*	Pathogenic	IEVA	Nl	Nl
XT089	F	8	c.IVS7-2A>G	Alteration of splicing sites	Pathogenic	c.2007C>G	p.D669E	Pathogenic	IEVA	Nl	Nl
XT042	M	12	c.IVS7-2A>G	Alteration of splicing sites	Pathogenic	c.2168A>G	p.H723R	Pathogenic	MD	Nl	Nl
XT074	M	12	c.IVS7-2A>G	Alteration of splicing sites	Pathogenic	c.2168A>G	p.H723R	Pathogenic	MD	Nl	
XT126	F	17	c.1079C>T	p.A360V	Pathogenic	c.1079C>T	p.A360V	Pathogenic	MD	Nl	Nl
XT121	M	10	c.1229C>T	p.T410M	Pathogenic	c.2168A>G	p.H723R	Pathogenic	MD	Nl	Nl
XT155	F	14	c.1692_1693insA	FS565, P573*	Pathogenic	c.2168A>G	p.H723R	Pathogenic	MD	Nl	Nl
XT047	M	11	c.147C>G	p.S49R	Pathogenic				Nl	Nl	Nl
XT033	F	10	c.IVS7-2A>G	Alteration of splicing sites	Pathogenic				IEVA	Nl	Total T3 higher
XT075	M	16	c.1472T>C	p.I491T	Pathogenic				Nl	Nl	Nl
XT133	F	20	c.1595G>T	p.S532I	Pathogenic				Nl	Nl	Nl
XT056	M	16	c.IVS16-6G>A	Alteration of splicing sites	Pathogenic				Nl	Nl	Nl
XT085	M	16	c.IVS16-6G>A	Alteration of splicing sites	Pathogenic				Nl	Nl	Nl
XT131	F	20	c.IVS16-6G>A	Alteration of splicing sites	Pathogenic	c.IVS11+47T>C	Alteration of splicing sites	Polymorphism	Nl	Nl	Nl
XT032	M	9	c.1764_1765 insAGGAAAATA	Frameshift	Pathogenic				Nl	Nl	Nl
XT001	F	6	c.2009T>C	p.V670A	Unkown				Nl	Nl	Nl
XT107	M	18	c.2009T>C	p.V670A	Unkown				Not done	Not done	Not done
XT090	F	14	c.IVS7-18T>G	Alteration of splicing sites	Polymorphism				Nl	Nl	Nl
XT076	F	18	c.IVS7-18T>G	Alteration of splicing sites	Polymorphism				Nl	Nl	Nl
XT013	M	8	c.IVS7-18T>G	Alteration of splicing sites	Polymorphism				Nl	Nl	Nl
XT079	M	22	c.IVS7-18T>G	Alteration of splicing sites	Polymorphism	c.IVS11+47T>C	Alteration of splicing sites	Polymorphism	Nl	Nl	Nl
XT015	F	10	c.1167G>A	p.G389G	Silent varients				NL	NL	Nl
XT091	F	11	c. IVS11+47T>C	Alteration of splicing sites	Polymorphism	c.IVS11+47T>C	Alteration of splicing sites	Polymorphism	Nl	Nl	Nl
XT058	F	13	c. IVS11+47T>C	Alteration of splicing sites	Polymorphism	c.IVS11+47T>C	Alteration of splicing sites	Polymorphism	Nl	Nl	Nl
XT069	M	6	c. IVS11+47T>C	Alteration of splicing sites	Polymorphism	c.IVS11+47T>C	Alteration of splicing sites	Polymorphism	Nl	Nl	Nl
XT043	M	9	c. IVS11+47T>C	Alteration of splicing sites	Polymorphism	c.IVS11+47T>C	Alteration of splicing sites	Polymorphism	Nl	Nl	Nl
XT088	M	11	c.IVS11+47T>C	Alteration of splicing sites	Polymorphism				Nl	Nl	Nl
XT007	F	13	c.IVS11+47T>C	Alteration of splicing sites	Polymorphism				Nl	Nl	Nl
XT009	F	15	c.IVS11+47T>C	Alteration of splicing sites	Polymorphism				Nl	Nl	Nl
XT017	F	7	c.IVS11+47T>C	Alteration of splicing sites	Polymorphism				Nl	Nl	Nl
XT027	M	9	c.IVS11+47T>C	Alteration of splicing sites	Polymorphism				Nl	Nl	Nl
XT035	M	20	c.IVS11+47T>C	Alteration of splicing sites	Polymorphism				Nl	Nl	Nl
XT161	M	18	c.IVS11+47T>C	Alteration of splicing sites	Polymorphism				Nl	Nl	Nl
XT050	F	15	c.IVS11+47T>C	Alteration of splicing sites	Polymorphism				Nl	Nl	Nl
XT061	F	7	c.IVS11+47T>C	Alteration of splicing sites	Polymorphism				Nl	Nl	Nl
XT095	F	9	c.IVS11+47T>C	Alteration of splicing sites	Polymorphism				Nl	Nl	Nl
XT100	M	10	c.IVS11+47T>C	Alteration of splicing sites	Polymorphism				Nl	Nl	Nl
XT106	M	11	c.IVS11+47T>C	Alteration of splicing sites	Polymorphism				Nl	Nl	Nl
XT119	M	13	c.IVS11+47T>C	Alteration of splicing sites	Polymorphism				Nl	Nl	Nl
XT120	F	14	c.IVS11+47T>C	Alteration of splicing sites	Polymorphism				Nl	Nl	Nl
XT124	F	20	c.IVS11+47T>C	Alteration of splicing sites	Polymorphism				Nl	Nl	Nl
XT140	F	11	c.IVS11+47T>C	Alteration of splicing sites	Polymorphism				Nl	Nl	Nl
XT143	M	10	c.IVS11+47T>C	Alteration of splicing sites	Polymorphism				Nl	Nl	Nl
XT156	M	15	c.IVS11+47T>C	Alteration of splicing sites	Polymorphism				Nl	Nl	Nl
XT157	M	20	c.IVS11+47T>C	Alteration of splicing sites	Polymorphism				Nl	Nl	Nl
XT163	F	9	c.IVS11+47T>C	Alteration of splicing sites	Polymorphism				Nl	Nl	Nl
XT006	F	10	c.IVS11+47T>C	Alteration of splicing sites	Polymorphism				Nl	Nl	Nl
XT160	F	12	c.IVS11+47T>C	Alteration of splicing sites	Polymorphism				Nl	Nl	Nl
XT070	M	14	c.IVS11+47T>C	Alteration of splicing sites	Polymorphism				Nl	Nl	Nl
XT103	M	15	c.1790T>C	p.L597S	Polymorphism				Nl	Nl	Nl
XT116	M	9	c.2283A>G	p.T761T	Polymorphism				Not done	Not done	Not done
XT104	F	11	c.2283A>G	p.T761T	Polymorphism				Nl	Nl	Nl
XT105	F	12	c.IVS11+47T>C	Alteration of splicing sites	Polymorphism				Nl	Nl	Nl

Abbreviations: IEVA, isolated enlarged vestibular aqueduct; MD, Mondini dysplasia; TBCT, temporal bone CT; TBUS, thyroid B ultrasound; TF, thyroid function; Nl, normal; F, female; M, male.

*, termination codon.

### CT scan

**Figure 1:**
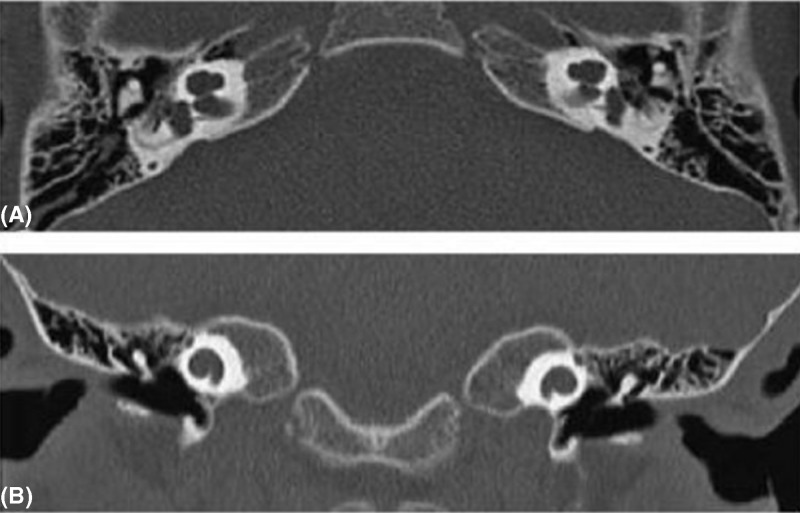
High-resolution temporal bone CT examination of the 63 patients with SLC26A4 mutation revealed MD in 15 patients CT image examples: (**A**) axial and (**B**) coronal.

**Figure 2:**
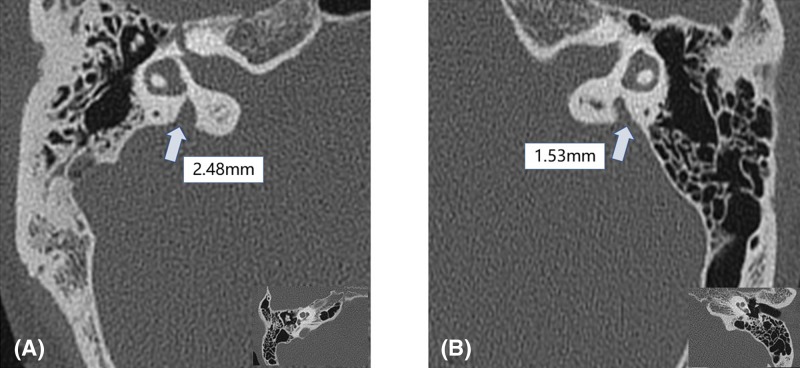
High-resolution temporal bone CT images of 2 cases with SLC26A4 mutation revealed IEVA Two cases ((**A**) patients XT148 and (**B**) XT089) of high-resolution temporal bone CT image; examples from 5 patients with IEVA in 63 patients with SLC26A4 mutation, based on the criteria of diameter greater than 1.5 mm between the outer of vestibular aqueduct and the vestibule of the total port foot or the midpoint of the isthmus.

**Figure 3:**
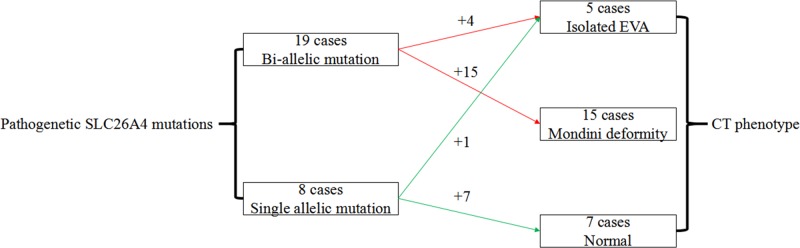
Schematic illustration of relationship between SLC26A4 mutations and CT phenotype The 100% of bi-allelic SLC26A4 mutation can lead to inner ear malformation including 3 cases of IEVA and 12 cases of MD in this cohort, while only 1 of 8 cases of single allelic mutation resulted in IEVA.

High-resolution temporal bone CT examination of the 63 patients with SLC26A4 mutation revealed isolated EVA (IEVA) or Mondini dysplasia/deformity (MD) in 20 patients. A total of 15 patients of the 20 patients had MD (Figure 1), while the other 5 patients had IEVA (Figure 2). It was indicated that all 15 patients who had MD were caused by bi-allelic loss of function of pendrin protein, 4 of 5 patients carried IEVA were caused by bi-allelic mutation, while the other one was caused by single pathogenic mutant allele (Figure 3). Additionaly, the most frequent mutation, IVS7-2 A>G, can lead to finding 100% of patients with IEVA (3 cases) and Mondini deformity (12 cases) in this cohort.

### Thyroid ultrasound and thyroid hormone assays

Thyroid ultrasound was performed to determine presence or absence of goitre. As shown in Table 1, none of the patients with bi-allelic SLC26A4 mutations or variants was diagnosed with goitre. Only one patient (patient XT033) with IEVA was found with a slightly elevated total T3 thyroid hormone assays, but there was no significant change in thyroid size found by ultrasound scan.

### Correlation of CT phenotype with age and gender of pathogenetic SLC26A4 mutation carriers

**Figure 4:**
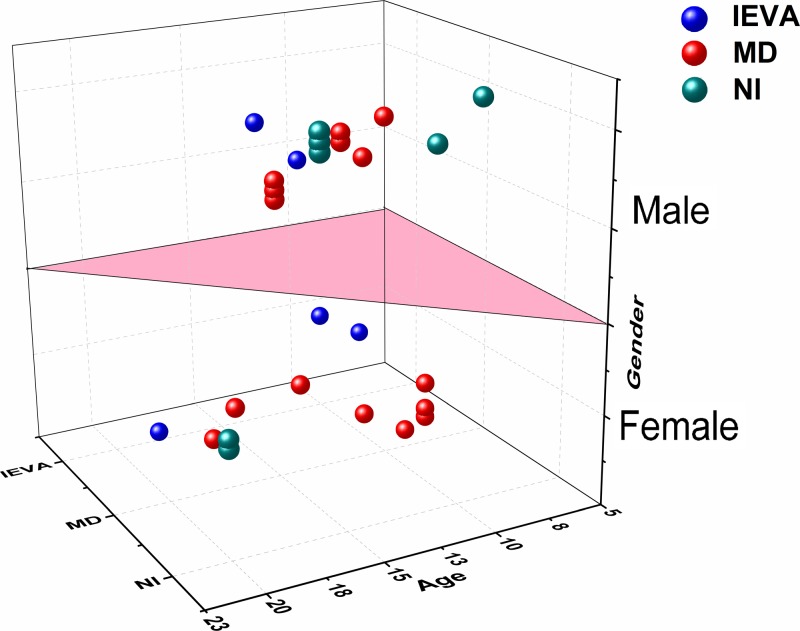
Correlation between CT phenotype, age, and gender of 27 patients with pathogenetic SLC26A4 mutation in 155 deaf children in Xiamen There is no significant statistical difference between average age of IEVA (12 years) and MD (12.2 years) (*P*>0.1). And also there was no gender difference in this disease (female/male = 13/14). Nl, normal; IEVA, isolated enlarged vestibular aqueduct; MD, Mondini dysplasia.

The average age of 20 patients (20/27) with EVA or MD was 12.15, while the average age of onset of the other patients (7/27) was 15.4 years. There is no significant statistical difference between the two groups (*P*>0.1). As shown in Figure 4, the detection rate of IEVA in pathogenetic SLC26A4 mutations carriers was 18.5% (5/27), and that of MD and normal were 55.6% (15/27) and 25.9% (7/27), respectively. However, there was no significant difference in gender (female/male = 13/14) and age (average age of EVA: 12, MD: 12.2, normal: 15.4).

## Discussion

It has long been known that abnormally EVA may accompany congenital malformations of the cochlea and semicircular canals. Recently, enlargement of the vestibular aqueducts as the sole radiographically detectable inner ear anomaly has been recognized as a distinct pattern of congenital inner ear malformation. Pathogenesis of the large vestibular aqueduct syndrome probably stems from an early derangement in the embryogenesis of the endolymphatic duct. This anomaly appears to be relatively common in children with SHL and is probably significantly underdiagnosed. Hearing loss is typically bilateral and progressive, with stepwise rather than fluctuant hearing decrements often triggered by relatively minor head trauma [19]. At present, the diagnosis of the large vestibular aqueduct mainly depends on the high-resolution CT examination of the temporal bone. However, in China, the cost of a temporal CT scan is more than 300 RMB (approximately 3 days’ salary of an average Chinese) and the CT examination needs the patients who are present and can co-operate correctly. Because of the limitations, it is not possible to perform a CT scan in every hearing loss patient for diagnosis of EVA.

Genetic diagnosis of deafness has a unique advantage in the etiological diagnosis of sensorineural deafness. In particularly, the *SLC26A4* gene mutation has been proved to be a true cause for a considerable number of deaf children [20]. For some children who are unable to accept or co-operate with CT examination or some sensorineural patients who are difficult to diagnose by CT, gene detection can assist or improve the diagnostic level.

In some Asian studies, the detection rate of *SLC26A4* mutations in patients with EVA (with or without MD) were 87, 97.9, 92, and 78% in Taiwanese, Chinese, Japanese, and Korean EVA patients, respectively [21–23]. Meanwhile, the mutation detection rate of this gene in Caucasian EVA patients is much lower, at 53 and 40%, respectively, in the U.K. and Europe [8,24]. In our study, we found that 100% patients (19 patients) carrying *SLC26A4* biallelic mutations had bilateral IEVA or MD. These findings confirmed *SLC26A4* mutations in hearing loss patients indicate a high possibility of EVA. So a striking achievement of the present study is the establishment of a new strategy for detecting *SLC26A4* mutations prior to temporal bone CT scan for identifying patients with EVA. This model has the unique advantage of identifying patients with EVA in an epidemiologic study in large-scale deaf populations.

During fetal development, the vestibular aqueduct starts out as a wide tube. By the fifth week it narrows, and by midterm it approaches adult dimension and shape, however, the vestibular aqueduct continues to grow and change until a child is 3–4 years old. As yet incompletely understood genetic or environmental conditions cause EVA, which is often congenital (present at birth) or occurs during early childhood [5]. However, there is no article that clearly points out the specific age of onset. Recently, many researchers suggested that patients with EVA have only one allelic mutant of the *SLC26A4* gene. In our study, only one patient carrying one allelic mutant of the SLC26A4 gene showed IEVA or MD. Zhao et al.’s [25] study revealed that copy number variations (CNVs) and the exon deletion in *SLC26A4* may be important factors in non-syndrome EVA (NSEVA). So that genome-wide studies to explore CNVs within non-coding regions of the *SLC26A4* gene and neighboring regions are warranted in order to elucidate their roles in NSEVA etiology.

PDS is known to be caused by homozygous or compound heterozygous mutations in the *SLC26A4* gene. The incidence of PDS is estimated to be as high as 7.5 to 10 in 100000 individuals, thus accounting for up to 10% of all hereditary hearing loss [26]. Whereas the phenotype of the Chinese PDS gene mutation is different from that of the European and American population [9,12,22]. In our study, 19 patients with homozygous or compound heterozygous mutations had no goitre. Their thyroid functions were also roughly normal that there was no characteristic of PDS. These may be explained by: (i) Our detection methods were different where we did not perform a perchlorate discharge test. (ii) The average age of patients who undertook thyroid ultrasound and thyroid hormone assays was 12.26 years in the present study, which may be too young to have symptoms, because goitre’s prevalence is approximately 73% in PDS and appears most commonly in the second decade of life. (iii) Phenotypic diversity due to different genetic backgrounds. Additionally, it is thought that the variability of the thyroid phenotype is, at least in part, influenced by the nutritional iodine intake. Subsequently, goitre development and thyroid dysfunction were barely detected in patients with bi-allelic *SLC26A4* mutations in our study, except one patient had slight elevation of free T3, since they came from Xiamen, a coastal city, with a high iodine intake diet.

## Conclusion

In the present study, we performed *SLC26A4* gene mutation analysis in combination with inner ear imaging on deaf patients at Xiamen City Special Education School and found 12.9% (20/155) had MD and IEVA. It was found that 100% patients (19 patients) carrying SLC26A4 biallelic mutations had bilateral IEVA or MD; 100% patients with IEVA carried *SLC26A4* mutations. These findings confirmed *SLC26A4* mutations in hearing loss patients indicate a high possibility of EVA or inner ear malformation. It was suggested that CT detection and gene detection can complement each other to improve the diagnosis of SHL. Moreover, because of a high iodine intake, goitre dysplasia and thyroid dysfunction were usually not observed in patients with biallelic mutations of *SLC26A4* from Xiamen.

## Abbreviations

CNV: copy number variation
CT: computerized tomography
EVA: enlarged vestibular aqueduct
IEVA: isolated EVA
MD: Mondini dysplasia/deformity
NSEVA: non-syndrome EVA
PDS: Pendred syndrome
SHL: sensorineural hearing loss
